# Physical activity calorie expenditure (PACE) labels in worksite cafeterias: effects on physical activity

**DOI:** 10.1186/s12889-019-7960-1

**Published:** 2019-11-29

**Authors:** Christopher B. Deery, Derek Hales, Laura Viera, Feng-Chang Lin, Zhaopei Liu, Emily Olsson, Julie Gras-Najjar, Laura Linnan, Seth M. Noar, Alice S. Ammerman, Anthony J. Viera

**Affiliations:** 1 Department of Family Medicine, University of North Carolina at Chapel Hill School of Medicine, 590 Manning Drive, Chapel Hill, NC 27599 USA; 2 Department of Health Behavior, University of North Carolina at Chapel Hill Gillings School of Global Public Health, Chapel Hill, NC USA; 3 NC Translational and Clinical Sciences Institute, University of North Carolina at Chapel Hill, Chapel Hill, NC USA; 4 Department of Biostatistics, University of North Carolina at Chapel Hill Gillings School of Global Public Health, Chapel Hill, NC USA; 5 Department of Nutrition, University of North Carolina at Chapel Hill Gillings School of Global Public Health, Chapel Hill, NC USA; 6 Sheps Center for Health Services Research, University of North Carolina at Chapel Hill, Chapel Hill, NC USA; 7 School of Media and Journalism, University of North Carolina at Chapel Hill, Chapel Hill, NC USA; 8 Center for Health Promotion and Disease Prevention, University of North Carolina at Chapel Hill, Chapel Hill, NC USA; 9 Department of Family Medicine and Community Health, Duke University School of Medicine, 2200 West Main Street, Suite 400, Durham, NC 27705 USA

**Keywords:** Calorie labeling, Physical activity, Obesity prevention policy, Worksite health promotion

## Abstract

**Background:**

Regular physical activity is an important component of healthy living and wellbeing. Current guidelines recommend that adults participate in at least 150 min of moderate or vigorous-intensity physical activity weekly. In spite of the benefits, just over half of U.S. adults meet these recommendations. Calorie-only food labels at points of food purchase have had limited success in motivating people to change eating behaviors and increase physical activity. One new point of purchase approach to promote healthy behaviors is the addition of food labels that display the physical activity requirement needed to burn the calories in a food item (e.g. walk 15 min).

**Methods:**

The Physical Activity Calorie Expenditure (PACE) Study compared activity-based calorie-expenditure food labels with calorie-only labels at three Blue Cross and Blue Shield of North Carolina worksite cafeterias. After 1 year of baseline data collection, one cafeteria had food items labeled with PACE labels, two others had calorie-only food labels. Cohort participants were asked to wear an accelerometer and complete a self-report activity questionnaire on two occasions during the baseline year and twice during the intervention year.

**Results:**

A total of 366 study participants were included in the analysis. In the PACE-label group, self-reported physical activity increased by 13–26% compared to the calorie-only label group. Moderate-to-vigorous physical activity (MVPA) increased by 24 min per week in the PACE-label group compared to the calorie-label group (*p* = 0.06). Changes in accelerometer measured steps, sedentary time, and MVPA had modest increases. Change ranged from 1 to 12% with effect size values from 0.08 to 0.15. Baseline physical activity level significantly moderated the intervention effects for all physical activity outcomes. Participants in both label groups starting in the lowest tertile of activity saw the largest increase in their physical activity.

**Conclusion:**

Results suggest small positive effects for the PACE labels on self-reported and objective physical activity measures. Minutes of weekly MVPA, strength training, and exercise activities showed modest increases. These results suggest that calorie-expenditure food labels may result in some limited increases in physical activity.

## Background

Regular physical activity is an important component of healthy living and wellbeing. People who are physically active generally live longer and have lower risk of adverse health outcomes [1]. The evidence supporting physical activity as an effective strategy in the primary and secondary prevention of chronic diseases as well as the reduction of all-cause mortality is compelling [2–5]. Current guidelines recommend that adults participate in at least 150 min a week of moderate-intensity or 75 min a week of vigorous aerobic physical activity [6]. In spite of the benefits, just over half of U.S. adults meet these recommendations [7]. Self-reported barriers to physical activity include lack of time, low motivation, or “no energy” [8–10].

Changing physical activity behavior is difficult [11, 12]. Multifaceted and leveled tactics are thought to be the most effective long-term strategies to increase physical activity [11, 12]. One promising novel approach is the addition of labels that display the amount of physical activity required to burn calories of selected food items [13]. The goal of these point of purchase food labels is to motivate consumers to increase physical activity or “nudge” them to reduce caloric intake [14]. Calorie information alone on food labels is unlikely to motivate people to change eating behaviors and increase physical activity [15–18]. According to behavioral economics theory, people will default to using mental shortcuts for many common decisions because our ability to process information is limited [19, 20]. In most cases, particularly when consuming meals not prepared in the home, people’s food-related decisions are not a function of rational processes. Approaches that rely on rational, reflective, or cognitive processes, such as reading and interpreting calorie information on food items, are unlikely to be effective. Several factors serve as barriers in making rational choices in this setting. Time pressures related to food breaks and distractions at points of purchase (such as a fast food restaurant line) are common. Interpreting calorie-only food labels during this limited interaction is challenging. Approaches that appeal to the intuitive system are much more likely to be effective. Intuitive approaches that are easily understood and interpreted and can happen quickly to compete with time pressures and distractions may be more effective in fostering behavior change. Activity-based food labels may be a more intuitive option for the consumer, offering a tangible “real-life” scenario as to what is actually required to burn the calories in food items (e.g. walking distance) [13]. It is thought that armed with this information, consumers may be influenced to make healthier eating choices and increase daily physical activity [13, 16].

The purpose of this paper is to examine changes in physical activity in participants exposed to activity-based food labels compared to those seeing standard calorie-only labels in their worksite cafeteria. Both self-report and objective physical activity data (accelerometer) were collected on study participants and are reported here.

## Methods

### Study design and population

This analysis is part of a larger two-group interrupted time series cohort study that examined the effects of an innovative food labeling system on calories purchased and levels of physical activity. In partnership with Blue Cross and Blue Shield of North Carolina (BCBSNC), the physical activity calorie expenditure (PACE) study compared activity-based calorie-expenditure food labels with calorie-only labels at three worksite campus cafeterias serving over 3600 employees. The study was approved by the Institutional Review Board of the Office of Human Research Ethics at the University of North Carolina at Chapel Hill.

A detailed description of the study design has been previously published [13]. In short, study participants were recruited in 2015 and were eligible for inclusion if they were (1) current BCBSNC employees or long-term contractors, and (2) intended to eat lunch in one of the campus cafeterias at least three times per 5-day work week. Participants were recruited through a combination of passive and active methods. Paper and electronic flyers advertising the study were placed throughout BCBSNC campuses and displayed on digital monitors. Study coordinators also actively recruited participants in the worksite cafeterias by setting up informational tables for employees to visit and to learn more about or sign up for the study. Cohort participant enrollment continued on a rolling basis throughout the baseline year to help compensate for attrition [13].

At the enrollment visit, participants met individually with study coordinators in a private room on the BCBSNC campus. Study coordinators explained the details of the study and obtained the participants’ informed consent and HIPAA waiver. All participants were asked to complete initial questionnaires with self-reported demographic items, medical and dietary history, and physical activity assessment forms on an electronic tablet. Cohort participants were also asked to wear an accelerometer and complete a self-report physical activity questionnaire on two occasions during the baseline year.

After the one-year pre-intervention (“baseline”) data collection, one cafeteria had food items prominently labeled with PACE activity-based labels and the other two cafeterias had food items prominently labeled with calorie-only labels. The two cafeterias receiving the calorie-only labels were combined to ensure that the number of participants and baseline demographic characteristics were similar to those of the cafeteria receiving the PACE labels. There was low concern for possible cross-over of participants at the cafeterias due to short lunch breaks and long distance between the various sites. It is unlikely that a study participant would have the time or ability to travel to any of the participating cafeterias other than the participant’s “home” cafeteria.

**Fig. 1:**
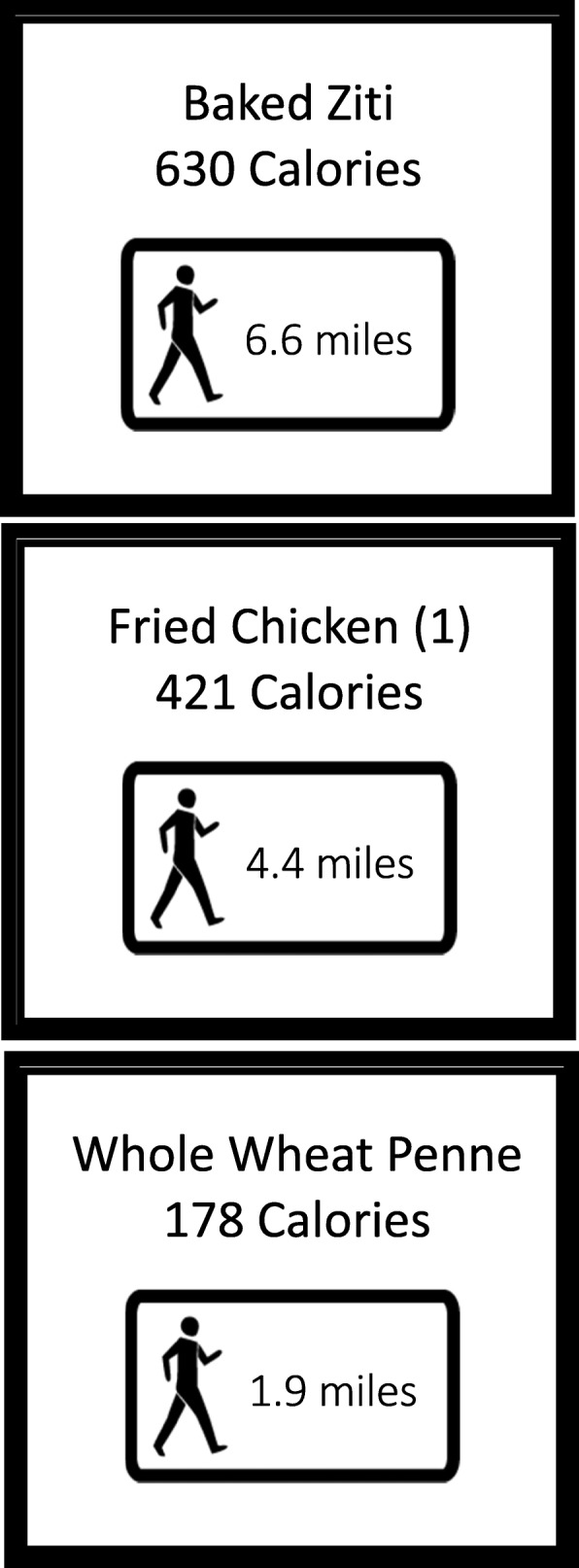
Example PACE labels

In the participating cafeterias, for all food prepared to order at the grill and deli, lists of commonly purchased items were posted with the PACE or calorie label. For salad bars, lists were posted of common items as well as representative salads showing the sum of calories or PACE equivalent from all ingredients included. Beverage cooler doors were labeled with lists of every beverage inside that specific door. Significant effort was made to ensure that the cafeterias served the same items during weeks when data collection was taking place. The same food company provides food to all 3 cafeteria sites, using the same products and recipes. During weeks without data collection, individual labels were removed, while deli list, grill list, salad list and representative salads and comprehensive beverage lists were left in place. All labels (PACE and calorie-only) measured 3 × 4 in. and were either bright green, bright blue, or bright yellow. The study team re-posted the labels in a different color than previously used at the beginning of each quarterly data collection period. The PACE labels were a focus group-tested image of a unisex figure walking with the number of minutes an average person would need to walk to burn the calories contained in the food item selected. An example PACE label is shown in Fig. 1. Follow-up measurements (accelerometer and self-reported questionnaire) occurred at two time points during year 2, while the labeling was in effect. Of note, our previous analysis found no difference in the efficacy of the PACE labels in reducing lunchtime calories purchased in worksite cafeterias compared with calorie-only labels [21].

### Measurements

Study participants were asked to complete a modified version of the CHAMPS physical activity questionnaire [22, 23] at four separate time points, two during the baseline year and two during the intervention year. Given that physical activity may vary considerably over the course of a year depending on weather, season, or other factors, participants were administered the questionnaire during the same months in the baseline year and intervention year to maintain consistency in calendar timing. Participants were asked to report the number of times, total minutes, and intensity of 27 common physical activities performed in the past week. Physical activities ranged from light household activities to intense conditioning and exercise. Outcomes were computed using minutes reported and standardized MET values for all moderate or vigorous activities. Secondary outcomes were estimated using a subset of 15 conditioning, exercise, and sport related activities. Baseline self-report data were averaged to estimate year 1 activity levels. Reports during the intervention year were averaged to estimate year 2 activity.

Objective estimates of physical activity were computed from data collected at four timepoints using the ActiGraph wGT3X-BT accelerometer (Fort Walton Beach, FL). Participants wore the accelerometer during the same months in the baseline year and intervention year for consistency. For logistical reasons, this wear did not directly overlap with the self-report but was generally within a few weeks. Participants were asked to wear the accelerometer at the right hip on a belt or clip for 7 days during each measurement period. Study coordinators provided instructions on how to wear the device. After measurement, data were downloaded and processed using Actilife (Actigraph, Inc.) and SAS V9.4. Wear and non-wear times were evaluated using the Choi algorithm [24] with additional removal of sleep and non-wear using logs and visual inspection of data. Daily minutes in sedentary, light, and moderate-to-vigorous physical activity (MVPA) were computed using established cut-points for adults [25]. MVPA bout minutes per day were also computed using a 10+ minute bout criteria. Participants needed 4 or more 8h hour days of wear to have outcomes computed. Baseline accelerometry data were averaged to estimate year 1 activity levels and data from the intervention year were averaged to estimate year 2 activity. On average participants wore monitors for 6.6 (1.2) days at each time point during year 1 and 6.3 (1.4) days each time during year 2. Participants averaged 13.9 (1.5) and 13.3 (1.5) hours of waking wear per day during year 1 and 2. Outcomes were standardized to a 14-h wear day to account for differences in total wear time among participants.

Demographics were self-reported via electronic questionnaire at study enrollment. Participants were included in this analysis if they had self-report or objective physical activity estimates for the baseline or intervention year.

### Statistical analysis

Standard descriptive statistics including means, percentages, and standard deviations were used to describe the study population. The primary analysis compared physical activity before and after the labeling interventions and the difference between the two groups. Analyses were performed under an intent-to-treat assumption using linear mixed models (SAS PROC MIXED) with maximum likelihood estimation and unstructured covariance matrix. Primary models included effects for time, arm, time x arm interaction, and covariates (age, sex, race, education, and income). Secondary analyses were conducted to examine moderation effects for sex (male, female), race (African American, White/Asian/Other), education (high/tech, college+), income (< 50, 50–99,100+), and baseline physical activity level (tertiles). Tests of moderation effects were similar to the primary analysis with the addition of a time x arm x moderator interaction term. All analyses were performed using SAS Software, version 9.4 (SAS Institute Inc., Cary, NC).

## Results

A total of 414 individuals initially consented to be in the study. Of these, 366 participants had adequate physical activity data during year 1 or year 2 (either self-report, Actigraph, or both). The final sample for analysis included 144 in the PACE-label group and 222 in the calorie-only label group. At follow-up, self-reported physical activity data were obtained from 63% of participants with 43% having complete accelerometer data. The sample was predominantly female (78%) with a mean age of 42 years, and mean BMI of 32 kg/m^2^ (Table 1). The demographics of the cohort reflected those of the entire employee population (3600 employees: 76% female, mean age of 43, BMI of 32 kg/m^2^). At baseline participants took approximately 5000 steps per day, were sedentary ~ 10 h per day, and were getting 20 min of MVPA per day. Differences between the intervention groups are highlighted in Table 1. In short, the calorie-only label group had more female participants (81% vs. 72%), more Caucasian participants (51% vs. 40%), fewer overweight/obese (78% vs. 86%), and fewer college graduates (62% vs. 70%).

**Table 1:** Characteristics of the cohort at baseline comparing intervention groups

	Entire Sample (*n* = 366)	PACE-Label Group (*n* = 144)	Calorie-Label Group (*n* = 222)	*p*-value
Strength training (SD) (times per week)	1.4 (2.0)	1.2 (1.9)	1.5 (2.0)	0.27
MVPA minutes (SD) (per week)	201.5 (194.8)	195.5 (184.5)	205.4 (201.5)	0.65
Exercise MVPA minutes (SD) (per week)	113.3 (138.9)	104.7 (126.9)	118.9 (146.3)	0.36
Steps (SD) (per day)	5172 (1980)	4805.8 (1617.7)	5411.0 (2155.7)	0.01
Sedentary minutes (SD) (per day)	613.0 (52.8)	620.7 (49.1)	608.0 (54.6)	0.06
MVPA minutes (SD) (per day)	18.9 (13.6)	18.0 (12.5)	19.6 (14.2)	0.34
MVPA bout minutes (SD) (per day 10+ min bouts)	9.8 (12.8)	8.8 (12.2)	10.5 (13.1)	0.28
Age (SD) (years)	42.2 (10.2)	40.9 (9.6)	43.0 (10.5)	0.06
Female, %	77.6	72.2	81.1	0.05
Race, %				0.05
White	46.2	39.6	50.5	
Black	43.2	45.1	41.9	
Asian	5.7	9.0	3.6	
Other	4.9	6.3	4.1	
Hispanic ethnicity, %	4.9	6.9	3.6	0.15
Mean BMI (SD)[*n* = 274]^a^	31.8 (8.0)	32.1 (7.6)	31.6 (8.2)	0.60
Underweight, % (< 18.5)	0.4	1.0	0.0	0.18
Normal Weight (18.5–24.9)	19.0	13.3	22.2	
Overweight (25.0–29.9)	29.2	30.6	28.4	
Obese (> 30.0)	51.5	55.1	49.4	
Household income, %				0.32
< $49,999	30.3	27.8	32.0	
$50,000–$99,000	36.1	34.0	37.4	
$100,000 or more	33.6	38.2	30.6	
Education, %				0.20
High school	12.3	11.8	12.6	
Tech school/Assoc. deg.	23.0	18.8	25.7	
College graduate	37.4	43.8	33.3	
Master’s degree+	27.3	25.7	28.4	

^a^BMI was only available for 274 participants (98 PACE-Label, 176 Calorie-Label)

*p*-value for difference between PACE-Label and Calorie-Label groups at baseline

A summary of the intervention effects on physical activity can be found in Table 2. In the PACE-label group, self-reported physical activity increased by 13–26% compared to the calorie-only label group. Effect sizes were small (0.14–0.20 mean change in standard deviation units). Moderate-to-vigorous exercise activity increased by 24 min per week compared to the calorie-only label group (*p* = 0.06). Race was found to moderate self-reported MVPA and exercise minutes per week, with African Americans in the PACE-label group reporting a larger increase in minutes per week compared to other groups (Table 3).

**Table 2:** Means, standard deviation, percent change and effect size for PACE and Calorie-Label groups

Outcome	PACE-Label Group			Calorie-Label Group			Group Change		*P*-value for intervention effect (GRP x YEAR)		*P*-value for moderator effects (GRP x YEAR x Moderator)				
	Year 1	Year 2	% Diff	Year 1	Year 2	% Diff	% Diff chg	Effect size	NO COV	With COV	Sex	Educ	Inc	Race	PA tertiles
Self-Report															
Strength training (times per week)	1.2 (1.9)	1.5 (2.0)	24.6	1.5 (2.0)	1.4 (1.9)	−1.7	26.3	0.20	0.16	0.18	0.28	0.18	0.04	0.21	< 0.001
MVPA minutes (per week)	195.5 (184.5)	218.0 (220.6)	11.6	205.4 (201.5)	201.2 (188.2)	−2.1	13.3	0.14	0.11	0.09	0.70	0.17	0.31	0.004	< 0.001
Exercise minutes (per week)	104.7 (126.9)	124.4 (152.1)	18.8	118.9 (146.3)	115.0 (139.9)	−3.3	20.9	0.17	0.063	0.058	0.57	0.24	0.31	0.006	< 0.001
Accelerometer															
Steps (per day)	4816 (1628)	4841 (1892)	0.5	5411 (2168)	5190 (1919)	−4.1	4.8	0.12	0.10	0.11	0.68	0.197	0.197	0.164	< 0.001
Sedentary minutes (per day)	620.7 (49.5)	620.0 (55.9)	−0.1	607.7 (54.9)	614.8 (44.6)	1.2	−1.3	−0.15	0.10	0.10	0.56	0.74	0.035	0.075	< 0.001
MVPA minutes (per day)	18.0 (12.6)	18.7 (14.6)	3.7	19.6 (14.3)	19.2 (13.7)	−2.2	5.8	0.08	0.29	0.34	0.095	0.38	0.14	0.37	0.074
MVPA bout minutes (per day)	8.8 (12.3)	9.2 (12.2)	4.2	10.6 (13.1)	9.8 (12.2)	−7.5	11.8	0.09	0.30	0.28	0.16	0.47	0.20	0.26	0.001

Year 1: baseline year; Year 2: intervention year (PACE labels displayed)

%Diff = ((Y2 – Y1)/Y1)*100

Effect Size = ((PACE Y2 – PACE Y1) – (Calorie Y2 - Calorie Y1))/(Pooled std. Y1)

%Diff Change = (PACE %Diff) - (Calorie %Diff)

With Covariates (COV): age, sex, race, education, and income

**Table 3:** Means, percent change, and *p*-values for moderators of the PACE label intervention effects

Outcome	Moderator	PACE-Label Group				Calorie-Label Group				
		Y1	Y2	% change	*p*-value	Y1	Y2	% change	p-value	Better impact
Self-Report										
Self-Reported Strength Training (times per week)	Income									
	Less than $50,000	2.05	1.93	-5.9	0.69	1.52	2.09	37.5	0.03	Calorie
	$50,000-$99,000	1.24	1.70	37.1	0.10	2.01	1.74	-13.4	0.23	PACE
	$100,000 or more	1.40	1.83	30.7	0.13	2.12	1.86	-12.3	0.27	PACE
	Starting PA level									
	Low (0 times)	0.22	0.77	250.0	0.02	0.23	0.71	208.7	0.01	Similar
	Mid (0.5-1.5)	1.33	1.53	15.0	0.59	1.26	1.16	-7.9	0.79	PACE
	High (2 or more)	3.85	3.48	-9.6	0.23	3.84	3.03	-21.1	0.004	PACE
Self-Reported MVPA^a^ (minutes per week)	Race									
	White, Asian, Other	237.6	202.9	-14.6	0.09	248.0	234.7	-5.4	0.38	Calorie
	African American	206.0	276.3	34.1	0.001	227.8	205.0	-10.0	0.25	PACE
	Starting PA level									
	Low (<80)	31.6	77.7	145.9	0.07	32.4	73.3	126.2	0.05	Similar
	Mid (80-233)	151.4	169.3	11.8	0.43	141.3	152.4	7.9	0.60	PACE
	High (234+)	418.4	385.6	-7.8	0.19	429.2	325.5	-24.2	0.0001	PACE
Self-Reported Exercise (Minutes per week)	Race									
	White, Asian, Other	112.4	91.3	-18.8	0.20	136.9	117.7	-14.0	0.13	Calorie
	African American	101.4	158.8	56.6	0.001	113.2	107.3	-5.2	0.71	PACE
	Starting PA level									
	Low (<30)	2.4	32.8	1266.7	0.13	-4.9	25.3	616.3	0.10	PACE
	Mid (30-129)	67.7	103.3	52.6	0.08	64.9	77.5	19.4	0.45	PACE
	High (130+)	246.0	217.1	-11.7	0.16	269.8	185.6	-31.2	0.0001	PACE
Accelerometer										
Steps (per day)	Starting PA level									
	Low (<4017)	3428	3502	2.2	0.80	3355	3719	10.8	0.20	Calorie
	Mid (4017-5559)	4625	5055	9.3	0.09	4713	4687	-0.6	0.93	PACE
	High (5560+)	7002	5950	-15.0	0.01	7691	6708	-12.8	0.0001	Similar
Sedentary (Minutes per Day)	Income									
	Less than $50,000	607	619	2.0	0.18	609	609	0.0	0.98	
	$50,000-$99,000	623	603	-3.2	0.02	618	627	1.5	0.20	PACE
	$100,000 or more	617	619	0.3	0.88	588	599	1.9	0.15	PACE
	Race									
	White, Asian, Other	624	607	-2.7	0.02	609	613	0.7	0.48	PACE
	African American	608	615	1.2	0.27	601	614	2.2	0.08	PACE
	Starting PA level									
	Low (<598)	554	568	2.5	0.20	549	580	5.6	0.0001	PACE
	Mid (598-637)	616	604	-1.9	0.13	618	621	0.5	0.70	PACE
	High (638+)	666	658	-1.2	0.26	664	641	-3.5	0.008	Calorie
Moderate and Vigorous (Minutes per Day)	Sex									
	Male	27.2	30.5	12.1	0.20	26.0	29.5	13.5	0.17	Similar
	Female	14.6	14.3	-2.1	0.84	16.7	14.6	-12.6	0.08	PACE
	Starting PA level									
	Low (<10.9)	7.5	10.0	33.3	0.26	7.6	8.1	6.6	0.83	PACE
	Mid (10.9-20.5)	14.7	16.0	8.8	0.54	15.5	15.8	1.9	0.87	PACE
	High (20.6+)	32.9	26.9	-18.2	0.02	35.1	31.6	-10.0	0.05	Calorie
Moderate and Vigorous in 10+ minute bouts (Minutes per day)	Starting PA level									
	Low (<1.9)	-0.2	4.1	2150.0	0.04	0.5	1.7	240.0	0.53	PACE
	Mid (1.9-9.6)	4.6	4.9	6.5	0.88	4.7	5.8	23.4	0.59	Calorie
	High (9.7+)	23.0	15.7	-31.7	0.002	23.1	17.8	-22.9	0.001	Calorie

^a^Minutes of moderate-to-vigorous physical activity according to self-report (> 150 min/week)

Changes in accelerometer measured steps, sedentary time, and MVPA were modest but in a positive direction. Change ranged from 1 to 12% with effect size values from 0.08 to 0.15. Steps per day did not change in the PACE-label group, but decreased in the calorie-only group by ~ 200 steps per day. MVPA increased in the PACE group by ~ 1 min per day compared to calorie-only group. Moderation analysis showed that men in both groups increased their MVPA minutes by about the same amount and that women decreased MVPA, but women in the PACE-group showed less of a decline (2% vs. 13%).

Baseline physical activity level significantly moderated the intervention effects for all physical activity outcomes (Table 3). Participants who started in the highest tertile of activity in both label groups decreased their activity from year 1 to year 2. Those starting in the lower tertiles increased their physical activity, with the middle tertile generally increasing but less than the lowest group. For accelerometer measured MVPA minutes, the percentage increase in physical activity for those in the middle (9% vs. 2%) and lowest (33% vs. 7%) tertile were larger for the PACE-label group compared to the calorie-only label group. Overall the interaction effects were more positive for the PACE-label group.

## Discussion

The purpose of this study was to examine changes in self-reported and objectively measured physical activity in people exposed to activity-based food labels and calorie-only food labels in a work site cafeteria setting. Results suggest small positive effects for the PACE activity-based labels on self-reported (~ 20% increase) and objective (~ 6% increase) physical activity. Minutes of weekly MVPA, step count, strength training, and exercise activities all slightly increased among participants who received the PACE labels compared with calorie-only labels although the differences observed were not statistically significant. Starting activity level was found to significantly moderate the intervention effects on all physical activity outcomes. To our knowledge this is the first study examining whether exposure to point-of-decision activity-based calorie-expenditure food labels translates to an increase in levels of physical activity.

Behavior change interventions such as point-of-decision prompts to increase physical activity have shown positive results [26]. Signs nudging individuals to use the stairs rather than taking the elevator to increase physical activity have had some success [27, 28]. Results of food labeling strategies (calorie-only and activity-based) to improve food choices and increase physical activity have been more varied. Exposure to labels with calorie information and physical activity equivalents at points of food purchase reduced the odds of buying sugar sweetened beverages among Black adolescents [29] and prompted parents to encourage exercise among their children [30]. Pilot data from participants who visited hypothetical fast food restaurants reported that they would select meals with fewer calories if shown activity-based food labels versus the traditional calorie-only labels [31]. Conversely, other studies have demonstrated that calorie-only menu labels in fast-food and restaurant settings are likely ineffective in encouraging lower calorie purchases [15–18]. Our previous analysis found that PACE labels were no more effective than calorie-only labels in reducing lunchtime calories purchased in the worksite cafeteria setting [21].

In this study, exposure to PACE labels had a modest positive effect on participant physical activity. Participants may have experienced a “nudge” from the labels that was sufficient to encourage transient increases in physical activity after exposure (e.g. taking stairs back to the office rather than the elevator), but not enough to have a large impact on MVPA. While important, these small daily changes are harder to measure and quantify.

The real-life effect of the PACE labels on physical activity may actually have been more than was reflected in our analysis due to several measurement limitations. Participants only wore the accelerometer during four periods throughout the study which may not have adequately captured activity changes in response to the food labels. In addition, some physical activity such as strength training and cycling would not be accurately reflected by the accelerometer. Other small, positive behavior changes such as participants standing at their desks or taking the stairs back to their offices would not have been quantified as increased MVPA.

Though the effect of the PACE labels on physical activity was small, these results are encouraging given the low-cost of the intervention and ability to scale it up. Calorie information on food items are already displayed on menu boards at restaurants with 20 or more locations as a result of the 2010 Patient Protection and Affordable Care Act. It would not be unreasonable to transition these food labels to more consumer-friendly, activity-based labels.

Certain factors may account for why the PACE labels did not have a larger impact. Time between exposure to the labels at lunch and when participants were free to exercise (e.g. before or after work) may have been too great. Information from the food labels may have been forgotten or the energy expenditure requirements no longer seemed a priority. Barriers to physical activity at work including time constraints, lack of energy, and limited flexibility [32] could have hindered any immediate change in habits. Confusion over the energy expenditure requirements for various food items or simply disregarding the labels are other possibilities.

We acknowledge that the effects on self-reported MVPA are subject to potential recall bias and over-reporting due to social desirability sometimes associated with this method [33]. But we also found that the objective accelerometer data support a similar small positive intervention effects on MVPA, which is encouraging. For the primary analysis all available data were used, but only 63% of participants had complete self-report data with only 43% having accelerometer data in both year 1 and year 2. Response rates were similar across intervention groups for self-reported physical activity. For the accelerometer data, the PACE group had a 10% higher response rate at year 2. While substantial efforts were made to collect complete data on all participants, loss to follow-up was substantial. To help account for missing data, maximum likelihood estimation was used in all models. In addition, the demographic makeup was similar for those with and without year 2 data. The one exception was for education level. Participants with year 2 data tended to have higher levels of education. About 70% had a bachelor’s degree or higher, but for those without year 2 data only 60% had a bachelor’s degree or higher. The worksite setting and high proportion of female participants may also limit the generalizability of our results. But the robust cohort design, diverse study population, two-year implementation, and the multiple measures of physical activity strengthen and support our findings.

While overall effects were modest, the results support the need for additional study of factors that may interact with intervention effects in important ways. Sex, race, and income show potential as moderators, not just covariates in this study. For example, women decreased MVPA in both groups but to a greater degree in the calorie-only group, while men increased MVPA in both groups. While the overall effect was not statistically significant and could be artifact, we should consider the potential that women and men respond differently to food labels. Of greater import is the strong and consistent interaction of starting physical activity level and change over time. More studies should be powered to test this interaction as a primary outcome and interventions should include it in exploratory analyses.

Refining this intervention could have a broader, more sustained effect. Providing consumers with additional prompts may strengthen their commitment to increase physical activity levels. An example would be sending a picture of the lunch food label by text after work hours or the next morning to remind them of their calories and the amount of activity associated with those foods. This contact may help to reinforce the point of purchase prompt and “nudge” them during a time more convenient for increased physical activity. Periodic prompts have been successful in producing positive results for short-term health behavior changes [34]. Personalizing or tailoring the labels based on feedback from participants may increase their efficacy. Another potential for the PACE label is to provide varying label information throughout the year. Changes in signage or point-of-decision prompts have been shown to have immediate impact that decay over time [35]. Periodic changes to cafeteria food labeling (size, color, design) may also “remind” people to notice them and act on the information provided.

## Conclusions

The small yet positive effects of this study suggest that calorie-expenditure food labels alone may result in some limited increases in physical activity. Given this, activity-based food labels could be considered as an adjunct tool in a broader behavior change strategy to increase physical activity. Coupling these labels with newer technologies and tactics, such as text prompts or goal setting for wearables (i.e FitBit, Apple health), may have a larger and more lasting effect. Inadequate physical activity is a modifiable risk factor for numerous adverse health outcomes and early death. Given the importance of physical activity in promoting health, continued development and evaluation of organizational level policy solutions such as calorie-expenditure food labeling is warranted.

## Data Availability

The datasets generated and analyzed during the current study are available from the corresponding author on reasonable request.

## Abbreviations

BCBSNC: Blue Cross Blue Shield of North Carolina
MVPA: Moderate-to-vigorous physical activity
PACE: Physical activity calorie expenditure
